# Neutrophil count in sputum is associated with increased sputum glucose and sputum L-lactate in cystic fibrosis

**DOI:** 10.1371/journal.pone.0238524

**Published:** 2020-09-11

**Authors:** Bibi Uhre Nielsen, Mette Kolpen, Peter Østrup Jensen, Terese Katzenstein, Tacjana Pressler, Christian Ritz, Inger Hee Mabuza Mathiesen, Daniel Faurholt-Jepsen

**Affiliations:** 1 Cystic Fibrosis Centre Copenhagen, Department of Infectious Diseases, Rigshospitalet, Copenhagen, Denmark; 2 Department of Clinical Microbiology, Rigshospitalet, Copenhagen, Denmark; 3 Costerton Biofilm Center, Institute of Immunology and Microbiology, Faculty of Health and Medical Sciences, University of Copenhagen, Copenhagen, Denmark; 4 Department of Nutrition, Exercise and Sports, University of Copenhagen, Frederiksberg, Denmark; University of Georgia, UNITED STATES

## Abstract

**Background:**

Markers of lung inflammation measured directly in expectorated sputum have the potential of improving the timing of antibiotic treatment in cystic fibrosis (CF). L-Lactate might be a marker of inflammation, as it is produced from glucose by polymorphonuclear neutrophils (PMNs) in CF lungs. We aimed to investigate changes in and associations between PMNs, glucose and L-lactate in sputum during antibiotic treatment. In addition, the effect of hemoglobin A1c and plasma glucose on these biomarkers were investigated.

**Methods:**

We sampled non-induced sputum at day 0, 7, 14 and 42 in 27 chronically infected CF patients electively treated with 14 days of intravenous antibiotic. To analyze sputum samples, we used flowcytometry to count PMNs and colorimetric assays to estimate lactate and glucose.

**Results:**

No changes in levels of PMNs, glucose and lactate were detected in sputum during the antibiotic treatment. Sputum PMNs were positively associated with both glucose (log coefficient = 0.20, p = 0.01) and L-lactate (log coefficient = 0.34, p<0.001). In multivariate analyses, hemoglobin A1c was negatively associated with sputum PMNs (log coefficient = -1.68, p<0.001) and plasma glucose was negatively associated with sputum glucose (log coefficient = -0.09, p = 0.02).

**Conclusions:**

In CF sputum PMNs, glucose and lactate were unchanged during elective antibiotic treatment. However, sputum PMNs were associated with both sputum glucose and sputum lactate. Surprisingly, hyperglycemia seemed to be associated with less PMNs infiltration and less glucose in CF sputum.

## Introduction

Frequent intravenous antibiotic treatments extend the life expectancy of patients with cystic fibrosis (CF) [1]. Intravenous antibiotics is used both for acute treatments of pulmonary exacerbations and for elective treatments to reduce chronic inflammation and lung function decline [2]. Yet, assessing the effect of treatment is challenged as the available tools are deficient. The most frequently used tool is spirometry [3, 4] despite its limited information on lung inflammation. Therefore, measuring biomarkers directly in sputum might be a superior approach for assessing lung inflammation.

Polymorphonuclear neutrophils (PMNs) are the predominant leukocytes in CF sputum during stable conditions [5, 6] and they become more abundant during pulmonary exacerbations [7, 8]. The endobronchial mucus of CF patients is hypoxic [9] and under anaerobic conditions PMNs derive energy by degrading glucose to L-lactate [10], as L-lactate is the predominant isotype produced by human cells [11].

A previous study in CF found higher sputum L-lactate during pulmonary exacerbations compared to post treatment [12]. This could be explained by numerous metabolic active PMNs during exacerbations. However, elevated levels of glucose in the lungs during exacerbations, similar to what is seen in chronic obstructive lung disease [13], could also contribute to increased L-lactate concentrations, as a result of extra energy substrate for the PMNs.

In a previous study, elevated levels of glucose were found in nasal airway surface liquid in patients with dysregulated CF related diabetes (CFRD) [14]. The authors suggested that there was a glucose spill over from plasma into the nasal airway liquids and that this might also apply to the lower airways. Thus, more substrate is provided to bacteria and inflammatory cells in the lungs, thereby potentially explaining the lung function decline related to CFRD [15]. However, the effect of CFRD on inflammation in CF lungs remain undetermined.

The aim of this study was to investigate sputum levels of PMNs, glucose and L-lactate before and after elective treatment with intravenous antibiotics in CF patients. Furthermore, we sought to describe the associations between PMNs, glucose and L-lactate in CF lung inflammation and to describe the influence of hemoglobin A1c (HbA1c) and plasma glucose on these potential biomarkers of inflammation.

## Methods

### Study design, setting and data collection

The study participants (≥ 18 years) were enrolled from the adult department at the Copenhagen CF Centre. We invited CF patients, who were chronically infected with gram-negative bacteria. As a standard of care in Denmark, chronically infected CF patients receive 14 days of intravenous treatment electively every 3 months. Patients treated as both out- and inpatients were invited to participate prior to a planned treatment. Gram-positive bacteria are not perceived as causing chronic infection and is usually not treated with intravenous antibiotics in Denmark. Thus, patients having only *Staphylococcus aureus* were therefore not included. Moreover, only patients capable of expectorating sputum spontaneously were eligible for inclusion, as tracheal suction material would be diluted and not useful for analyses.

The study was approved by Regional Committee on Health Research Ethics (Region H: H-16037693), and all enrolled patients signed a consent form. At the Copenhagen CF Centre, data from all patients about previous health, lung function, blood samples and lung microbiology are entered prospectively in a database where baseline data was extracted from.

### Sputum samples

Sputum samples were collected at day 0 (beginning of intravenous treatment), day 14 (end of intravenous treatment) and at day 42 (at the next routine consultation). If the patient was close to or admitted to the hospital, a sputum sample was also collected at day 7. The sputum samples were brought to the laboratory and prepared <5 hours after sampling. Judged by experienced laboratory staff, samples were excluded if they consisted solely of saliva; identified according to their high clarity and fluidity as well as lack of purulence. Furthermore, to avoid saliva in the samples, the most viscous and yellow/green expectorated sputum was collected in a syringe (303172; BD Bioscience) before diluting 0.2 ml sputum in 10 ml phosphate buffered saline (PBS), pH = 7.4 (Substrate Department, Panum Institute, Copenhagen, Denmark). The sputum samples were then resuspended by gentle pipetting. However, the material was not drained to eliminate traces of saliva.

### Neutrophil count

The concentration of neutrophils in sputum samples was determined by adding 100 μL diluted sputum to a TrueCount tube (BD 340334; Becton Dickinson). In addition, 5 μL of each of the following mouse anti-human monoclonal antibodies (BD Bioscience) were added to the samples: peridinin chlorophyll A protein-labelled CD14 PerCP (BD345786), FITC-labelled CD15 (BD5554019), and allophyocyanin (APC)-labelled CD45 (BD555485) (Bioscience, La Jolla, CA, USA). The samples were incubated for 30 min and fixated with 1 mL 1Xfluorescence activated cell sorter (FACS) lysis solution (BD349202) (Bioscience). Subsequently, the samples were stored overnight in the dark at 5°C before analysis by flow cytometry using a FACSCanto (Bioscience) equipped with a 15 mV argon-ion laser tuned at 488 nm, and a red diode laser emitting at 635 nm for excitation. Light-scatter and exponentially amplified fluorescence parameters from at least 10 000 events were recorded in list mode. Leukocytes were discriminated by gating on CD45 and the PMNs were discriminated by gating on CD14 and CD15. The instrument was calibrated using Calibrite beads (BD 349502, Bioscience) (S1 Fig).

The neutrophils were counted by flowcytometry as previously described [16] within 14 days.

### L-lactate and glucose measurements

L-lactate and glucose in sputum were assessed in duplicates using colorimetric assays according to the manufacturer’s instructions (Lactate Assay Kit (Sigma-Aldrich, Missouri, USA, # MAK064-1KT) and Glucose (GO) Assay Kit (Sigma-Aldrich, Missouri, USA, #GAGO20)). For L-lactate estimation, the standard curve solutions and 50 μL of sample suspension was mixed with 50 μL of Master Reaction Mix containing 4% Enzyme mix, 4% probe and 92% buffer. After 30 minutes incubation the fluorometric intensity was measured at 570 nm and concentrations were estimated by comparing sample absorbance with the standard curve absorbance. Lactate dehydrogenase was not removed from the samples.

For the glucose assessment we had to reduce sample volumes as well as kit substances to one tenth compared to the manufacturer’s instructions, as our samples were of small volumes. Standard curve solutions and 100 μL of sample suspension were all added 200 μL of assay reagent containing glucose oxidase and peroxidase and after 30 minutes the reaction was stopped by adding 200 μL of N 12 H_2_SO_2_. Concentrations were estimated by comparing sample absorbance with the standard curve absorbance at 540 nm. Spiked samples were not included in neither of the analyses.

### Lung capacity

Spirometry was conducted as a part of our routine procedures at day 0, day 14 and day 42. We used the JAEGER MasterScope PC (CareFusion, Hoechberg, Germany) for measurements in accordance with the ATS/ERS guidelines. Forced expiratory volume in 1 second percentage predicted (FEV_1_%) was obtained using the reference material provided by Global Lung Function Initiative (GLI) [17].

### HbA1c and plasma glucose

HbA1c was assessed at day 0 using a G8 HPLC Analyzer (Tosoh). HbA1c reflects a non-enzymatic glycosylation of hemoglobin during the last 120 days and only minor variation in HbA1c is likely to occur during a 42 days period in patients without changes in diabetes treatment or lifestyle [18]. Thus, only baseline HbA1c was used in the linear mixed models. Solely the first 10 patients enrolled had plasma glucose measured at every visit. Plasma glucose was derived from venous blood analyzed in a blood gas analyzer (ABL90 FLEX by Radiometer).

### Statistics

For the sample size calculation, we used previous data on levels of lactate in exacerbation and post-exacerbation sputum samples from CF patients with the mean (±SD) being 3.4 (± 2.3) and 1.4 (± 1.4) mmol/L, respectively [12]. Furthermore, we chose a power of 90% and a significance level at 5%. Based on this we needed 20 patients for our study. However, as our study patients were not having exacerbation, we anticipated a lower reduction in sputum lactate levels between day 0 and 14, and therefore we enrolled 7 additional patients during the 5-month data collection period.

We conducted data analyses in Stata 12. Right-skewed outcomes were logarithm transformed. Model checking was carried out using residual and Q-Q plots.

We used linear mixed models, which utilize all available data, to estimate time differences in biomarkers and associations between the biomarkers (sputum PMNs, sputum glucose, sputum L-lactate as well as HbA1c and plasma glucose). Furthermore, a linear mixed model including both sputum PMNs and sputum glucose was fitted to estimate the adjusted associations between these biomarkers and L-lactate. Similarly, we fitted linear mixed models including both HbA1c and plasma glucose for each sputum biomarker.

In the linear mixed models a random effect was assigned to each participant, as each participant contributed non-independent data from repeated samples. Day of sampling was also assigned a random effect in the models of biomarker correlation. We used the significance level of 0.05. Robust standard error was used in the linear mixed models to eliminate bias by heteroscedastic data and missing data was random in all variables.

## Results

### Baseline characteristics

We included 27 participants with CF from whom we had samples from day 0 and 14, with the exception of one patient who was unable to expectorate at day 14. Fourteen participants delivered sputum at day 7, and 11 participants delivered sputum at day 42. The total number of observations, missing values and extreme values are listed in the supplementary material (S1 Table). Twenty-four (88.9%) participants were chronically infected with *Pseudomonas aeruginosa*, 2 (7.4%) participants with *Burkholderia multivorans* and 1 (3.7%) participants with *Achromobacter xylosoxidans*. In total, 16 (59.3%) of the participants had a bacteria species sensitive to a least one of the chosen antibiotics, 3 (11.1%) had an intermediate sensitive species and 8 (29.6%) had a species resistant to the chosen antibiotics (Table 1).

**Table 1:** Baseline characteristics in the CF study population.

Characteristics	
Participants, n (%)	27 (100%)
Age, median (range)	39 (20–66)
Male, n (%)	18 (66.7%)
**Pathogen of chronic infection, n (%)**	
*Pseudomonas aeruginosa*	24 (88.9%)
*Burholderia multivorans*	2 (7.4%)
*Achromobacter xylosoxidans*	1 (3.7%)
**Sensitivity to antibiotic treatment during the study, n (%)**	
Sensitive	16 (59.3%)
Intermediate	3 (11.1%)
Resistant	8 (29.6%)
**Glucose tolerance†, n (%)**	
Normal	7 (25.9%)
Impaired	6 (22.2%)
Pathological (CFRD)	14 (51.9%)

† Glucose tolerance is based on 2-hour plasma glucose (mmol/L) in an oral glucose tolerance test: Normal <7.8, impaired ≥7.8 and <11.1 and pathological ≥11.1. CFRD, cystic fibrosis related diabetes.

**Fig 1:**
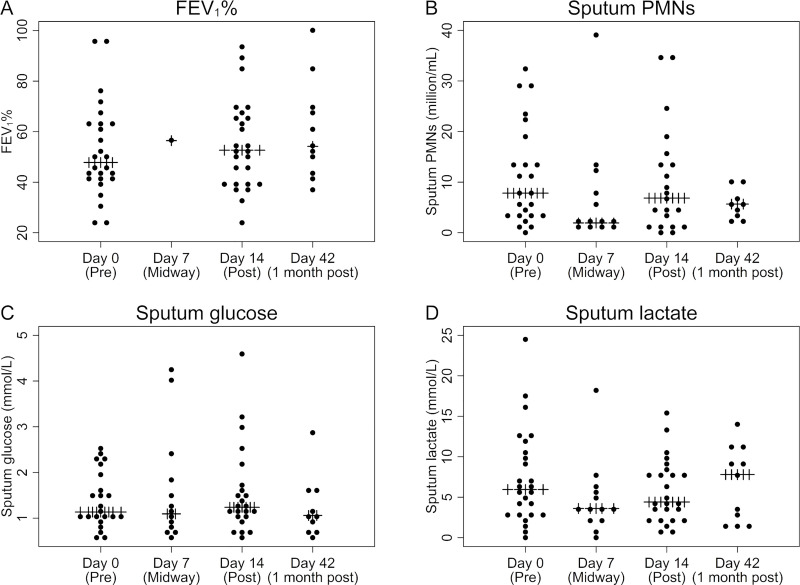
Lung function and sputum biomarkers during and after intravenous antibiotic treatment in CF patients. A) FEV_1_% (n = 26; 1; 25; 10) B) sputum PMNs (n (extreme values) = 26 (1); 13(1); 22(1); 9) C) sputum glucose (n(extreme values) = 27(4), 14(1), 26(3), 11(1)) and D) sputum L-lactate levels (n(extreme values) = 27(1), 14(1), 26(1), 11) during and after 14 days of intravenous treatment. Extreme values outside the outer fences (lower: Q1–3 IQR and upper: Q3 + 3 IQR) were not depicted. However, they were includeded in the S2 Fig and in the linear mixed models, which showed no significant difference between day 0 and 14 for FEV_1_% and log transformed measurments of PMNs, glucose and L-lactate in sputum. FEV_1_%, forced expiratory volume in 1 second percentage predicted. PMNs, polymorphonuclear neutrophils.

At baseline (day 0), the median FEV_1_% was 47.8% (range: 23.9% - 96.7%), which increased to 52.3% (range: 23.2% to 92.8%) after 14 days of treatment. Using linear mixed models, the FEV_1_% was unchanged between day 0 and day 14 (p = 0.3). Including all samples, the median level of sputum PMNs was 6.2 million/mL (range: 0.3–271), sputum glucose was 1.2 mmol/L (range: 0.5–32.7) and sputum L-lactate was 5.3 mmol/L (range: 0.2–69.7). Between day 0 and 14 of the antibiotic treatment there was a non-significant decline in sputum PMNs (median: 11.2 vs. 6.6 millions/mL, p = 0.3) (Fig 1B), in sputum glucose (median: 1.5 vs. 1.3 mmol/L, p = 0.9) (Fig 1C), and in sputum L-lactate (median: 6.1 vs. 4.7 mmol/L, p = 0.7) (Fig 1D). There was no association between the change in FEV_1_% and the change in PMNs from day 0 to day 14 (coefficient = -1.9, p = 0.5) (S3 Fig).

### Associations between PMNs, glucose and L-lactate in sputum

**Fig 2:**
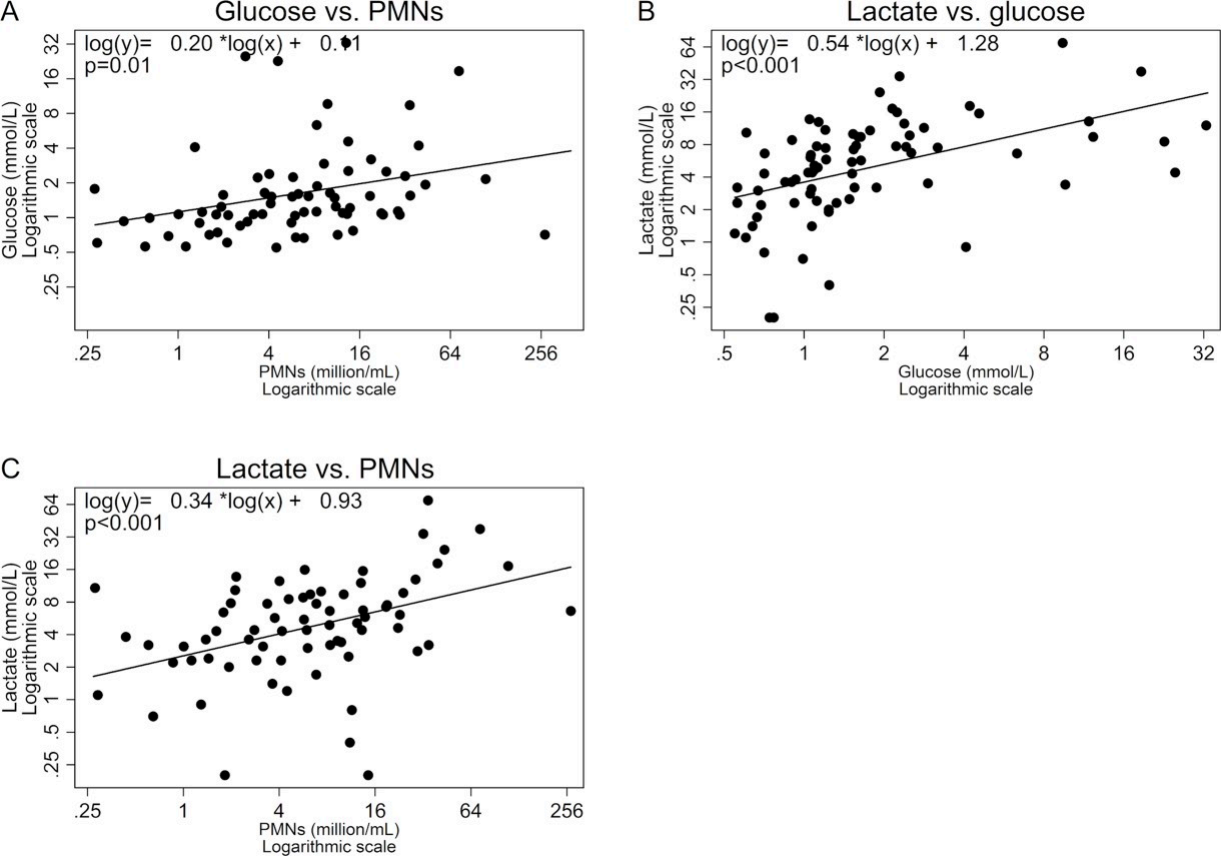
Associations between PMNs, glucose and L-lactate in sputum from CF patients. Associations between A) sputum PMNs and sputum glucose (n = 70), B) sputum glucose and sputum L-lactate (n = 78), C) sputum PMNs and sputum L-lactate (n = 70). Coefficients and lines are derived from linear mixed models accounting for non-independent data points. PMNs, polymorphonuclear neutrophils.

Using linear mixed models on transformed and pooled data from all sampling days, we found a positive association in sputum samples between PMNs and glucose (coefficient = 0.20, p = 0.01) (Fig 2A), between glucose and L-lactate (coefficient = 0.54, p<0.001) (Fig 2B), and between PMNs and L-lactate (coefficient = 0.34, p<0.001) (Fig 2C). When both PMNs and glucose were included in a multivariate analysis with sputum L-lactate the magnitude of both coefficients decreased but remained significant (coefficient for PMNs = 0.24, p = 0.014 and glucose = 0.43, p = 0.007).

### Associations between HbA1c, plasma glucose and sputum biomarkers

**Fig 3:**
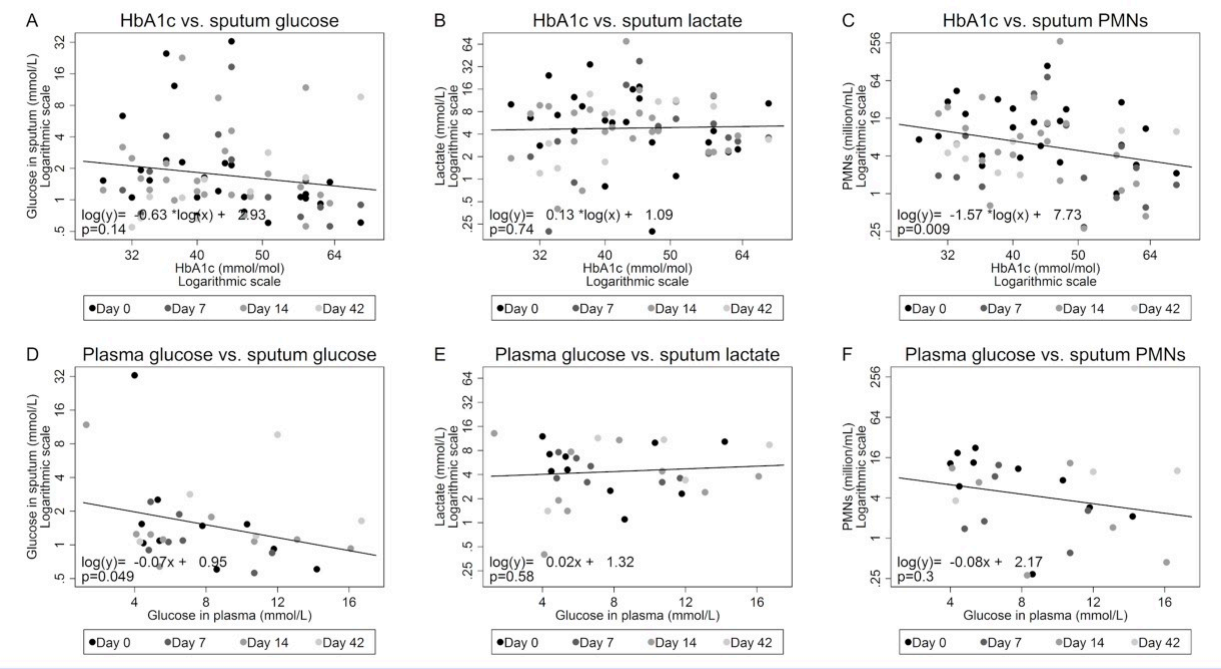
Hemoglobin A1c and plasma glucose compared to PMNs, glucose and L-lactate in sputum from CF patients. Associations between baseline (day 0) HbA1c and levels of A) sputum glucose (n = 75), B) sputum L-lactate (n = 75) and C) sputum PMNs (n = 68). Associations between plasma glucose and concurrent levels of D) sputum glucose (n = 31), E) sputum L-lactate (n = 31) and F) sputum PMNs (n = 25). Coefficients and lines are derived from linear mixed models accounting for non-independent data points sampled on different days during and after 14 days of intravenous antibiotic treatment. HbA1c, Hemoglobin A1c. PMNs, polymorphonuclear neutrophils.

In univariate linear mixed models on transformed and pooled data from all sampling days, baseline HbA1c (coefficient = -0.63, p = 0.14) (Fig 3A) was not associated with sputum glucose, but plasma glucose was negatively associated with sputum glucose (coefficient = -0.07, p = 0.049) (Fig 3D). After including both factors in a multivariate model the effect of plasma glucose on sputum glucose was unchanged (coefficient = -0.09, p = 0.018) (Table 2). Sputum L-lactate was neither associated with HbA1c at baseline (Fig 3B) nor plasma glucose at sampling (Fig 3E). However, we found a significant negative association between baseline HbA1c and sputum PMNs in the univariate analysis (coefficient = -1.57, p = 0.009) (Fig 3C), which remained significant in the multivariate analysis (coefficient = -1.68, p<0.001) (Table 2). No significant association could be detected between plasma glucose and sputum PMNs (Fig 3F and Table 2).

**Table 2:** Multivariate linear regressions with hemoglobin A1c and plasma glucose vs. different sputum biomarkers in CF patients.

	Multivariate					
	Sputum glucose		Sputum lactate		Sputum PMNs	
	Coefficient (95%-CI)	P-value	Coefficient (95%-CI)	P-value	Coefficient (95%-CI)	P-value
**HbA1c**	0.49 (-0.08–1.06)	0.09	0.34 (-0.84–1.52)	0.6	-1.68 (-2.28 - -1.08)	<0.001*
**Plasma glucose**	-0.09 (-0.1.6 - -0.02)	0.018*	0.01 (-0.06–0.07)	0.82	-0.05 (-1.19–0.09)	0.5

Data is computed in linear mixed models. By assigning a random effect for each individual the results were adjusted for non-independent data from repeated samples. Standard errors were estimated as robust.

* Significant at p<0.05. HbA1c, hemoglobin A1c.

To visualize our findings, we have outlined the observed associations in an overall research model (S4 Fig).

### Sensitivity analyses

The antibiotic sensitivity to the chosen treatment did not significantly alter the changes in biomarkers from day 0 to day 14. There was also no association between plasma PMNs and sputum PMNs (coefficient = 0.1, p = 0.2) as well as no association between plasma PMNs and sputum glucose (coefficient = 0.2, p = 0.5) and sputum L-lactate (coefficient = 0.3, p = 0.4).

The linear correlation between baseline sputum glucose and the change in both FEV_1_% and sputum PMNs between day 0 vs. day 14 were also investigated, but no significant association was found (coefficient = 0.58, p = 0.6 and coefficient = -12.1, p = 0.1, respectively).

The significant association between baseline HbA1c and PMNs did not change after adjusting for age (coefficient = -2.24, p<0.001).

## Discussion

Among adult CF patients who received a 14 days course of elective intravenous antibiotic treatment, we found that PMNs, glucose and L-lactate in sputum as well as FEV_1_% remained unchanged. Independent of sampling time, sputum PMNs were positively associated with glucose and L-lactate in sputum. There was also a positive association between sputum glucose and sputum L-lactate, after adjusting for sputum PMNs. In the adjusted analysis, plasma glucose was negatively associated with sputum glucose, meanwhile HbA1c was negatively associated with sputum PMNs.

To our knowledge no previous study has been published on PMNs, glucose or L-lactate in sputum during elective intravenous antibiotic treatment in CF adults. In contrast to our study, a study including 9 stable CF children with recent *P*. *aeruginosa* colonization found a 74% decline in PMNs density from pre- to post- systemic treatment [19]. However, our patients are likely to differ from children since they are chronically infected and thus might be less responsive to treatment. Correspondingly, Downey *et al*. described results resembling ours, as adult CF patients systemically treated for a pulmonary exacerbation only had a non-significant decline in PMNs [7].

The positive association between PMNs and L-lactate in our study is also found in a CF study with patients having pulmonary exacerbation [20] and previous CF studies have found FEV_1_% to be negatively associated to both PMNs [21] and L-lactate [12]. The association in this study indicates that PMNs is a key producer of L-lactate in CF lungs. Moreover, we speculated that during stable periods metabolic inactive PMNs predominates in CF lungs compared to periods of exacerbation. This might explain the insignificant change in pre- and post-treatment levels of sputum biomarkers in the current study.

Furthermore, our results showed an association between glucose and L-lactate in sputum. It has previously been shown that hyperglycemia elevate lactate efflux from epithelial cells in the CF airway [22]. Hence, high glucose content in CF airway might stimulate production of L-lactate by PMNs in a similar way. However, it could be speculated that the associations seen in this study are simply due to inflammation-related confounding if sputum PMNs, glucose and L-lactate are all independent biomarkers that are elevated because of lung inflammation.

CFRD studies by Brennan *et al*. have indicated that during hyperglycemia plasma glucose spill over into the airway liquids [14, 23]. This contrasts with our study showing that plasma glucose was negatively associated with sputum glucose. Comparable to our study, Sambeek *et al*. showed that high sputum glucose was more frequent among patients with a normal glucose tolerance compared to patients with CFRD. Sambeek *et al*. also reported concentrations of sputum glucose higher than 30 mmol/L [24] similar to our study. Thus, in some cases sputum glucose concentrations exceeded the concentration of glucose in plasma, suggesting that glucose is not solely allocated into sputum by simple diffusion. A non-CF study on pneumonia and bronchiectasis using positron-emission tomography (PET) scans and γ-scintigraphies found that glucose uptake by PMNs takes place post- lung migration in pneumonia. However, this did not apply to bronchiectasis [25], with more similarities to CF disease. Likewise, another study using PET found glucose uptake to be normal in CF lungs and there was no association between levels of sputum PMNs and glucose uptake [26]. Hence, there was no evidence of PMNs inducing influx of glucose in the lungs. Therefore, we have reasons to believe that high sputum glucose in our study is due to carriage by migrating PMNs, and that some of the sputum glucose in our study originate from the intracellular contents of PMNs. Insulin is in vitro shown to upregulate glucose transporters in PMNs [27]. Moreover, insulin also increase the adhesion and hence migration of PMNs to the site of infection through upregulation of ICAM-1 in endothelium cells [28]. We therefore speculate, that high insulin levels contribute to high concentrations of glucose containing PMNs in the lungs, which could explain the association between low plasma glucose and high sputum glucose seen in our study.

Non-enzymatic glycosylated proteins, similar to HbA1c, have been shown to inhibit the PMNs membrane fluidity possibly leading to reduced mobilization of the adhesion proteins [29]. Thus, reduced adhesion and migration proteins on both the PMNs and endothelium cells in diabetic patients could contribute to the negative association between HbA1c and sputum PMNs seen in our study.

Other studies have suggested that non-enzymatic glycosylated products cause increased cytoplasmic calcium levels potentially leading to dysfunction in the PMNs [30] and high plasma glucose levels might inhibit PMNs function by inhibiting the oxidative burst of these cells [31]. Hence, in the lungs of CF diabetic patients PMN activity might be reduced, potentially resulting in a higher incidence of exacerbation due to an advantageous milieu for the bacteria [32].

Our study was conducted in a relatively homogenous study population, with almost all patients being stable and chronically infected with *P*. *aeruginosa*. Thus, we expect limited bias due to different bacterial colonization and unstable lung inflammation.

However, a limitation in this study was the risk of sputum dilution by saliva during the oral passage. If this dilution varies much among the samples, this could affect the associations between systemic biomarkers and biomarkers found in sputum. However, we have selected only the thick colored mucus from our samples shortly after collection to limit bias from dilution. To further avoid dilution, we chose not to include tracheal suction and bronchoalveolar lavage, as such procedures use indeterminate quantities of saline.

We wanted to be able to compare our data to our previous results, why according to routine laboratory procedure, dehydrogenase was not removed before investigating L-lactate concentrations in sputum. Lactate dehydrogenase converts L-lactate to pyruvate and it is linearly correlated to L-lactate in sputum [12], why we may have underestimated L-lactate concentrations.

To further assess the effect of the antibiotic treatment, it would have been relevant to count bacteria load before and after treatment, as a supplementation to the change in FEV1%. However, this was not done since it is not routine in CF treatment monitoring.

In conclusion, our study shows that sputum glucose and sputum L-lactate are associated with PMNs in sputum. However, neither of these biomarkers changed significantly during elective intravenous antibiotic treatment, although all three markers declined. Furthermore, hyperglycemia seems to be associated with lower sputum PMNs, which could affect the CF lung function negatively. Since a relative insulin deficiency in CF might be a risk factor for dysfunctional PMNs, further studies on the effect of insulin therapy on sputum PMNs, glucose and L-lactate are wanted in CF.

## Supporting information

S1 Fig Strategy for identifying neutrophils in sputum by flow cytometry. Gate for exclusion of debris of events recorded according to staining of CD45 (A). Gate for selection of single cells (B). Gate for identification of neutrophils according to high staining for CD15 (FL1-A) and low staining for CD14 (FL3-A) (C).
(TIF)

S2 Fig Lung function and sputum biomarkers during and after intravenous antibiotic treatment in CF patients. A) FEV_1_% (n = 26; 1; 25; 10) B) sputum PMNs (n = 26; 13; 22; 9) C) sputum glucose (n = 27, 14, 26, 11) and D) sputum L-lactate levels (n = 27, 14, 26, 11) during and after 14 days of intravenous treatment. All extreme values are included in the plot by using a logarithmic y-axis.
(TIF)

S3 Fig Difference in FEV_1_% from day 0 to day 14 compared to the concurrent decline in PMNs. (TIF)

S4 Fig Research model based on correlation coefficients. Straight arrows represent path between factors. +) is a positive association and -) is a negative association.
(TIF)

S1 Table Summary of data. n, total number of complete observations. Missing, total number of missing observations. Extreme values, determined by outer fences: (Q1–3 IQR) and (Q3 + 3 IQR).
(DOCX)

S1 File Data. (XLS)
