# Supplementary figures and images for: Neutrophil count in sputum is associated with increased sputum glucose and sputum L-lactate in cystic fibrosis
Source: PLoS One. 2020 Sep 11;15(9):e0238524. doi: 10.1371/journal.pone.0238524 (PMC7485830; doi:10.1371/journal.pone.0238524)

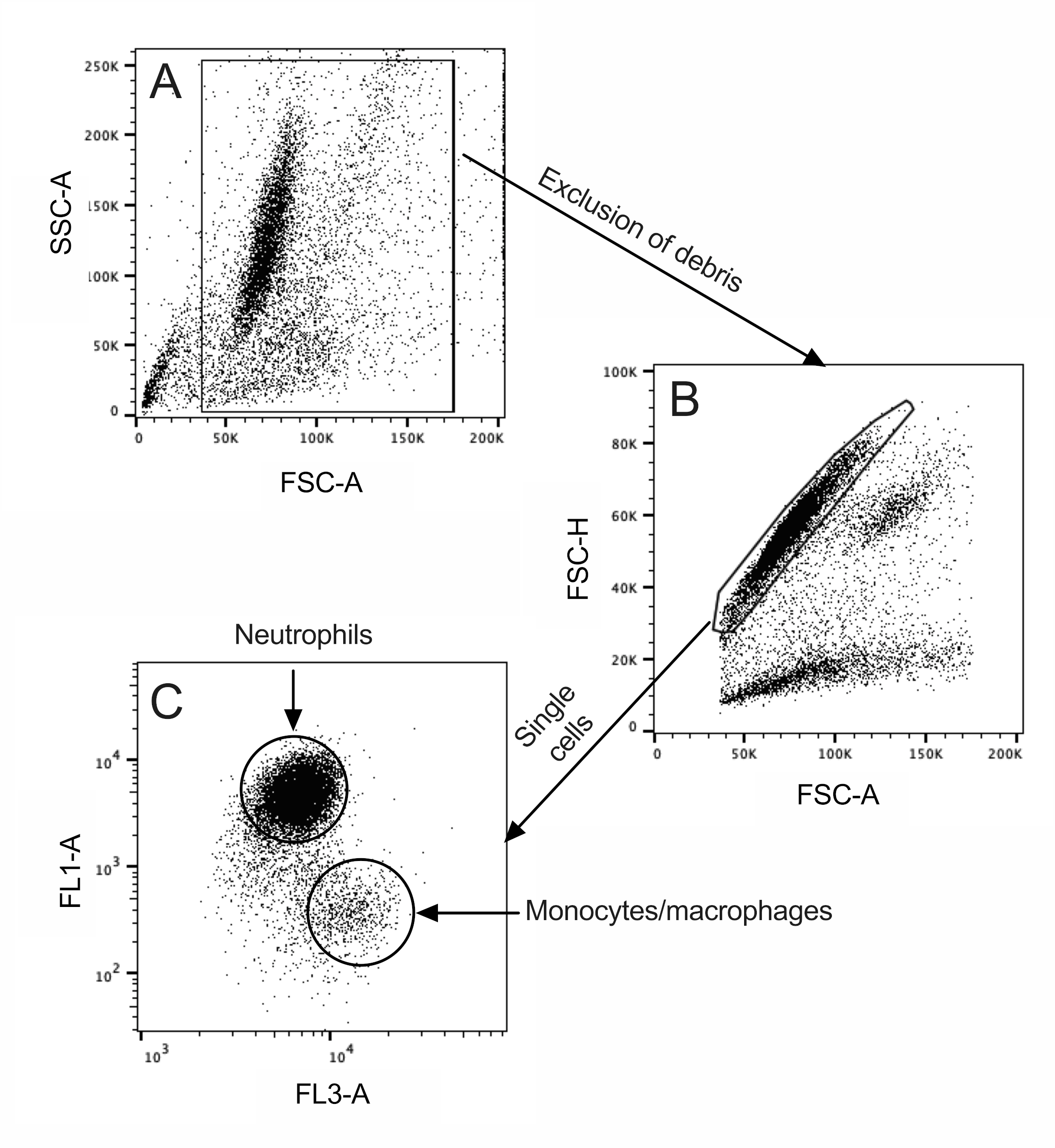

Supplement: S1 Fig — Gate for exclusion of debris of events recorded according to staining of CD45 (A). Gate for selection of single cells (B). Gate for identification of neutrophils according to high staining for CD15 (FL1-A) and low staining for CD14 (FL3-A) (C). (TIF) [file pone.0238524.s001.tif]

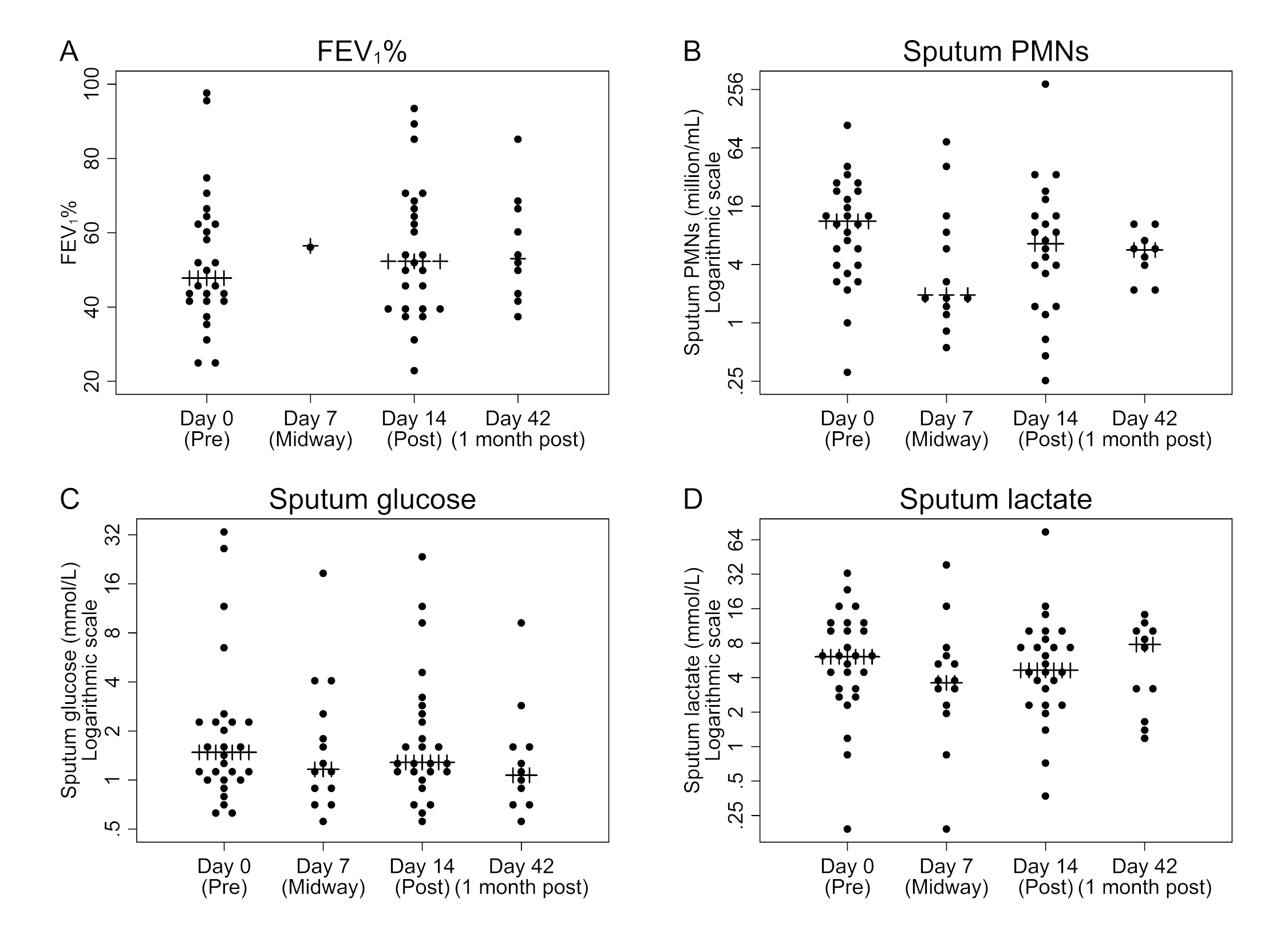

Supplement: S2 Fig — A) FEV1% (n = 26; 1; 25; 10) B) sputum PMNs (n = 26; 13; 22; 9) C) sputum glucose (n = 27, 14, 26, 11) and D) sputum L-lactate levels (n = 27, 14, 26, 11) during and after 14 days of intravenous treatment. All extreme values are included in the plot by using a logarithmic y-axis. (TIF) [file pone.0238524.s002.tif]

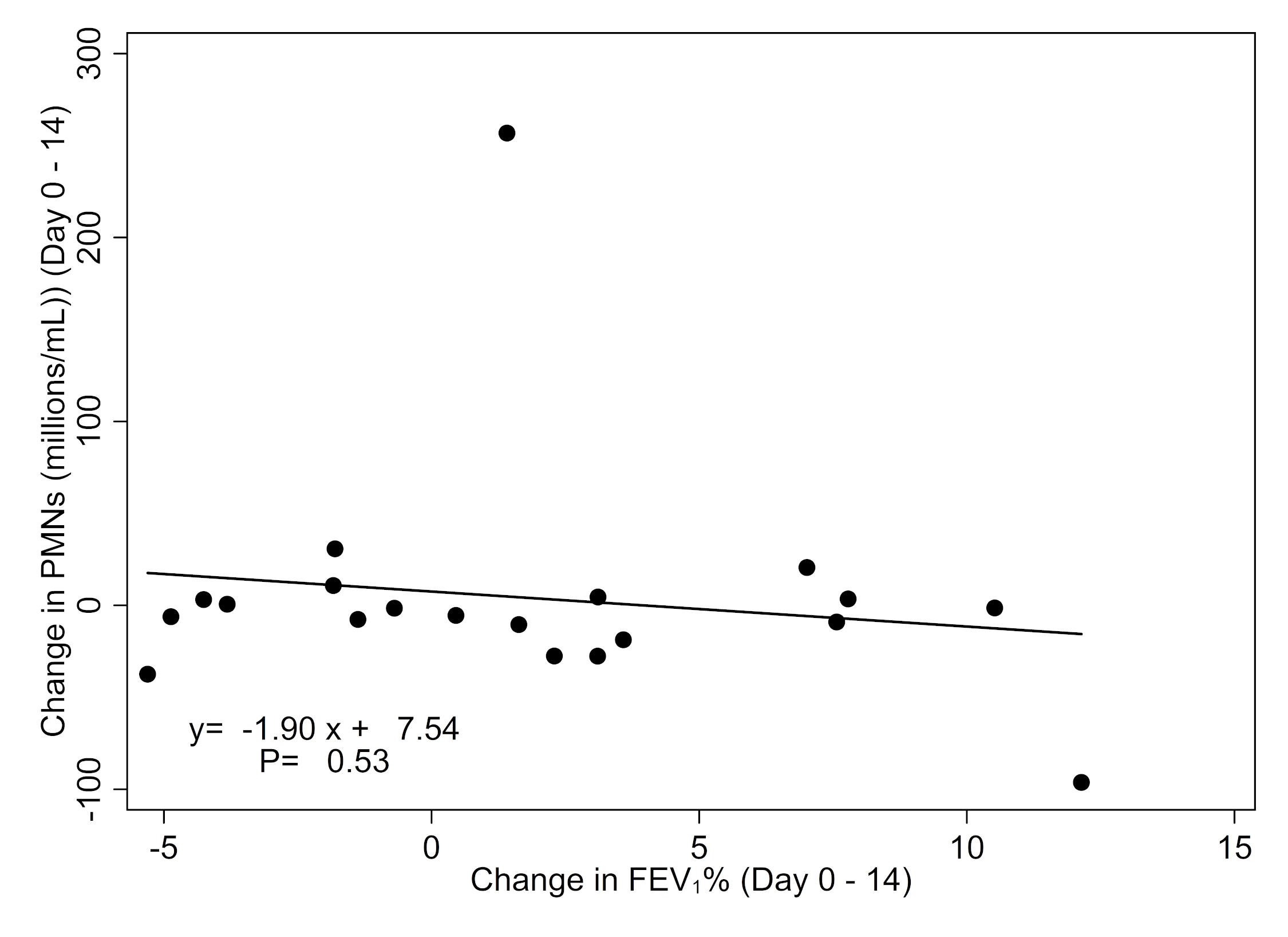

Supplement: S3 Fig — (TIF) [file pone.0238524.s003.tif]

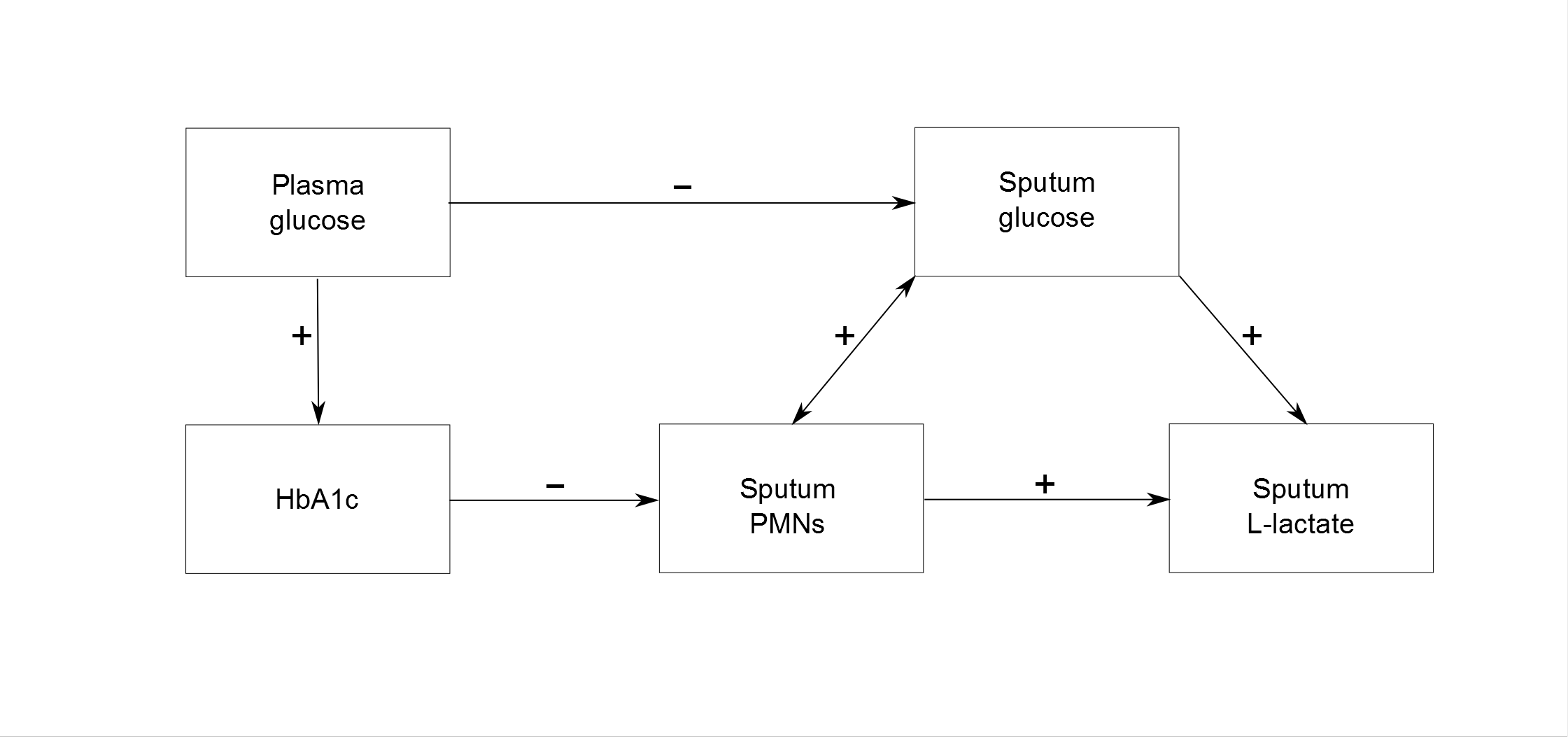

Supplement: S4 Fig — Straight arrows represent path between factors. +) is a positive association and -) is a negative association. (TIF) [file pone.0238524.s004.tif]
