# Supplementary material for: Neutrophil count in sputum is associated with increased sputum glucose and sputum L-lactate in cystic fibrosis
Source: PLoS One. 2020 Sep 11;15(9):e0238524. doi: 10.1371/journal.pone.0238524 (PMC7485830; doi:10.1371/journal.pone.0238524)
Supplement: S1 Table — n, total number of complete observations. Missing, total number of missing observations. Extreme values, determined by outer fences: (Q1–3 IQR) and (Q3 + 3 IQR). (DOCX) [file pone.0238524.s005.docx]

|  | All visits | | |  | Visit 1 (Day 0) | | |  | Visit 2 (Day 7) | | |  | Visit 3 (Day 14) | | |  | Visit 4 (Day 42) | | |
| --- | --- | --- | --- | --- | --- | --- | --- | --- | --- | --- | --- | --- | --- | --- | --- | --- | --- | --- | --- |
|  | n | missing | outliers |  | n | missing | outliers |  | n | missing | outliers |  | n | missing | outliers |  | n | missing | outliers |
| Sputum samples | 78 |  |  |  | 27 |  |  |  | 14 |  |  |  | 26 |  |  |  | 11 |  |  |
| FEV1% | 62 | 16 | 0 |  | 26 | 1 | 0 |  | 1 | 13 | 0 |  | 25 | 1 | 0 |  | 10 | 1 | 0 |
| Sputum PMNs | 70 | 8 | 3 |  | 26 | 1 | 1 |  | 13 | 1 | 1 |  | 22 | 4 | 1 |  | 9 | 2 | 0 |
| Sputum Glucose | 78 | 0 | 9 |  | 27 | 0 | 4 |  | 14 | 0 | 1 |  | 26 | 0 | 3 |  | 11 | 0 | 1 |
| Sputum L-lactate | 78 | 0 | 3 |  | 27 | 0 | 1 |  | 14 | 0 | 1 |  | 26 | 0 | 1 |  | 11 | 0 | 0 |
| HbA1c | 26 | 1 | NA |  | 26 | 1 | NA |  | NA | NA | NA |  | NA | NA | NA |  | NA | NA | NA |
| Plasma Glucose | 31 | 47 | NA |  | 10 | 17 | NA |  | 7 | 7 | NA |  | 9 | 17 | NA |  | 5 | 6 | NA |

*Table 1: Summary of data. n, total number of complete observations. Missing, total number of missing observations. Outliers, extreme values determined by outer fences: (Q1 – 3 IQR) and (Q3 + 3 IQR).*
